# Where Do Smokers in Singapore Smoke? A Latent Class Analysis to Classify Smokers Based On Smoking Location

**DOI:** 10.1093/ntr/ntaf076

**Published:** 2025-04-09

**Authors:** Zilu Feng, Chuen Seng Tan, Jeong Kyu Lee

**Affiliations:** Saw Swee Hock School of Public Health, National University Health System, National University of Singapore, Singapore, Singapore; Saw Swee Hock School of Public Health, National University Health System, National University of Singapore, Singapore, Singapore; Department of Health and Exercise Science, The University of Oklahoma, Norman, OK 73109, USA

## Abstract

**Introduction:**

A key lever protecting people from secondhand smoke (SHS) exposure is not sharing the same space as smokers while they smoke. However, there is limited research on where exactly smokers choose to smoke, particularly in urban multi-ethnic Asian contexts. This study aims to identify distinct classes of smokers in Singapore and characterize smokers based on where they smoke.

**Methods:**

Using responses from 1,546 participants in Wave 2 of the Singapore Smokers’ Survey (2021), latent class analysis identified distinct smoker classes based on predefined smoking location options reported for home and outside home in the past month. Multinomial logistic regression was conducted to examine differences between classes on various covariates.

**Results:**

Three distinct latent classes of smokers were identified: “smoke at work” (75.7%), “smoke outside home” (14.0%), and “smoke everywhere” (10.2%). Ethnicity, housing type, marital status, hardcore smoking indicator (yes vs no), smoking status (daily vs occasional), time taken to smoke the first cigarette of the day after waking and perceiving family norms towards smoking habit were significant predictors of class membership. “Smoke at work” was the reference class. Those who “smoke everywhere” were more likely to be Malay, hardcore smokers and smoked their first cigarette of the day within 30 minutes of waking.

**Conclusions:**

Observed heterogeneity amongst Singapore smokers supports implementation of more targeted interventions for both smoking cessation and protecting nonsmokers from SHS exposure. Social norm change strategies may be considered, leveraging upon the roles of familial influence and ethnicity on where smokers choose to smoke.

**Implications:**

This study identifies three distinct smoker subgroups based on smoking locations—at work, outside the home, and everywhere—offering actionable insights for targeted interventions. Workplace-based cessation efforts can focus on those smoking at work, while promoting smoke-free homes may address familial pressures in the outside-home group. For those smoking everywhere, intensive interventions are needed to reduce SHS exposure across settings. Multinomial regression reveals that familial disapproval, ethnicity, and housing type significantly influence smoking patterns, highlighting the importance of culturally tailored interventions that leverage social, familial, and environmental factors to reduce smoking prevalence and SHS exposure.

## Introduction

One key lever to protect the general population from the harms of secondhand smoke exposure (SHSe) is to not share the same space as smokers while they smoke, particularly in enclosed spaces with poor ventilation.^1^ Hence, banning smoking from various public places, especially indoor spaces has been a key pillar to tobacco control,^2^ and serves to shape social norms around cigarette use—signaling that smoking is frowned upon, and smoking in the presence of others is not socially acceptable.^3^ They can be particularly effective at shaping the macro-environment when strongly enforced, with significant impacts on SHSe and smoking prevalence.^4^ On the other hand, evidence suggests that prohibiting smoking in certain places have simply displaced the behavior to other locations,^5^ such as from public spaces to the home.

It is well established that homes and workplaces are among the most common locations for SHSe and where smokers typically smoke.^6^ However, research exploring specific locations of smoking, particularly within the home is limited. One study in Belgium found that the most frequently mentioned places for smoking at home by smokers is the living room and kitchen,^7^ and since these are shared spaces in the household, it would put other members of the household at greater risk of SHSe. Hence, a better understanding of where smokers smoke could be useful in understanding where the burden of SHSe is the greatest. In the same vein, a better understanding of the differences in behaviors of different types of smokers, in particular hardcore smokers who smoke frequently in large quantities, could also be useful in diagnosing the target groups of intervention who may be present a higher risk of exposing others to secondhand smoke (SHS).

Characterizing the types of smokers based on their likelihood to smoke in various locations can be useful in formulating and prioritizing smoking cessation or SHS reduction interventions and evaluating existing tobacco control policies. Since behaviors, norms, and regulations surrounding smoking are heavily influenced by social and environmental contexts, it would be valuable to examine these questions in Singapore, a highly urbanized, multi-ethnic country in Southeast Asia with a unique social setting distinct from much of the existing tobacco research. Singapore has one of the strictest tobacco control landscape having banned smoking in most public places (indoors and outdoors) and high rates of taxation.^8^ Smoking prevalence in Singapore fell from nearly 25% in the 1970s to approximately 11% in 2019.^9^ Singapore’s experiences could serve as a model for other rapidly developing countries in the region, offering insights for improving tobacco-related health outcomes and reducing SHSe in their populations.

This study will seek to better understand where smokers in Singapore smoke, both at home and outside the home setting. It will further seek to characterize smokers based on where they choose to smoke, including an interest in hardcore smokers. The study aims to use latent class analysis (LCA) to identify distinct classes (hidden subgroups or clusters) of smokers in Singapore by characterizing their preferred smoking locations within the past month; and to use multinomial logistic regression to determine how these identified classes differ on a variety of covariates such as sociodemographics, smoking-related characteristics, lifestyle and health status.

## Methods

### Study Design and Population

Data for this study was obtained from Wave 2 of the Singapore Smokers’ Survey (SSS)^,10^. Wave 1 of the SSS project served as the foundation for questionnaire development, ensuring consistency and comparability across waves.^11^ The survey was conducted between 1 July and 5 September 2021 as a follow-up to the SSS first conducted between 3 December 2019 and 2 May 2020, where participants were recruited by convenience sampling, starting with existing participants from the Singapore Population Health Studies (SPHS).^12^ For detailed information on SPHS, please see https://blog.nus.edu.sg/sphs/. Participants in these cohorts who consented to be re-contacted for future studies were invited for this study. This was further complemented by recruitment at designated smoking areas in public places around Singapore, flyer distribution and personal contacts. Participants of the SSS were required to be current smokers, defined as having smoked at least 100 cigarettes in his or her lifetime and currently smoking every day or some days.^13^ The survey was available in all four of Singapore’s official languages (English, Chinese Mandarin, Malay, and Tamil), and consent was taken prior to the commencement of the online survey. Ethics approval was obtained from National University of Singapore Institutional Review Board (NUS-IRB: S-19-230). The response rate of Wave 2 amongst SSS participants who had agreed to be re-contacted for future research was 85.4%, leading to a total of 1,873 respondents.

**Figure 1.**
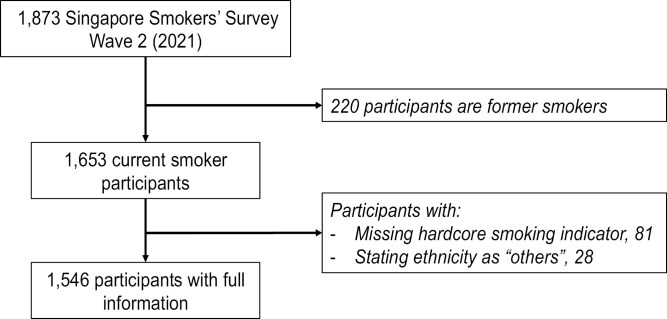
Study participants flow chart.

As the study is interested in where current smokers smoke, 220 respondents who had quit smoking since the first wave of SSS were excluded from the analysis. Given our further interest in characterizing where hardcore smokers may prefer to smoke and the role of ethnicity given the multi-ethnic nature of Singapore and the heterogeneity in the behavior of smokers across different ethnic groups, the small group who was unable to be assigned hardcore smoking status due to missing information and those who stated their ethnicity as “others” were excluded from the analysis. A total of 1,546 records were retained for analysis (Figure 1).

### Measures

#### Smoking Location At Home

Participants were asked “In a typical week in the past month, where did you smoke in your residence/home? (Select all that apply).” The response options were: “Living room,” “Kitchen,” “Toilet(s),” “Your bedroom,” “Balcony/garden/porch/garage,” “Other areas in your home” and “Don’t know / Not sure.” These variables were treated as binary indicator variables and coded as 1 when selected (and 0 otherwise). Some of the locations are specific to the Singapore context, while others were included with reference to existing studies.^7,14,15^

#### Smoking Location Outside Home

Participants in SSS Wave 2 were asked “In a typical week in the past month, where did you smoke outside your home? (Select all that apply).” The response options were: “Never smoked outside your home,” “At/near work,” “At/near schools/childcare centers/kindergartens/universities,” “At/near hospitals/clinics/polyclinics,” “At/near restaurants/hawker centers,” “At/near bars/clubs,” “At/near bus stops/MRT stations,” “At/near HDB corridors/staircase/void decks/carparks,” “At/near public parks/playgrounds,” “At/near religious venues,” “At/near community centers/facilities,” “In other persons’ home,” “In other persons’ vehicle,” “Public sidewalks/pavements,” “Others” and “Don’t know / Not sure.” These variables were treated as binary indicator variables and coded as 1 when selected (and 0 otherwise). Some of the locations are specific to the Singapore context, while others were included with reference to existing studies.^7,14,15^

#### Hardcore Smoking Status

To be characterized as a hardcore smoker, which is our key outcome variable, smokers had to meet the following 3 criteria: (i) responded with a value larger than 15 to “On average, about how many cigarettes do you smoke each day?,” (ii) responded “no” to “During the past 12 months, have you stopped smoking for one day (24 hours) or longer because you were trying to quit?” and (iii) responded “within the next 1 year,” “more than a year later,” or “no intention to quit” to “How soon are you likely to quit smoking?” This definition is used in numerous other studies and therefore allows comparison with international studies.^16,17^ All daily smokers who did not meet the criteria were considered non-hardcore smokers.

#### Smoking-related Characteristics

All participants were asked about their smoking behaviors. Besides questions used to identify current smokers, participants were also asked whether they smoked his or her first cigarette within half an hour of waking for the day.^18,19^ As a measure of the environments that smokers smoke in, such as social relationships and social norms around smoking behavior, participants were asked about the number of their closest friends whom they hang out with the most who also use tobacco (three categories: “none or less than half,” “approximately half,” “most or all”), self-perceived level of approval of their smoking habits by family and friends (dichotomized to “approve” vs “neutral/disapprove”), and whether they have discussed their desire to quit smoking with their family and friends in the past month (dichotomized to “yes” vs “no/not sure/don’t know”).

#### Sociodemographics

Sociodemographic variables included ethnicity as stated on national identification documents (three categories: “Chinese,” “Malay” or “Indian”), age (grouped into four categories: “19–24,” “25–44,” “45–64,” ≥65’), gender, level of highest education attained (four categories: “primary and below,” “secondary,” “pre-university,” “university and above”), current marital status (three categories: “currently married,” “never married,” “divorced/separated/widowed”), and type of residence (four categories: “HDB 1–2 room flat,” “HDB 3-room flat,” “HDB 4-room flat,” “HDB 5-room flat/executive flat/landed property/condominium/ others”), which is used as a proxy to socioeconomic status and/or income (almost 80% of Singapore population lives in public housing known as HDB (Housing Development Board) flats, which are rented or bought from the government, with eligibility and the amount subsidized determined by household income and size of apartment sought.^20^

#### Lifestyle and Health Status

Data were collected on participants’ lifestyle patterns and health status, including alcohol consumption (four categories: “non-drinker,” “occasional drinker,” “frequent drinker,” “regular drinker”), self-perceived health status (five categories: “excellent,” “very good,” “good,” “fair,” “poor”) and amount of weekly physical activities (four categories: “none,” “1–2 times,” “3–6 times,” “daily”).

### Data Analysis

All analyses were conducted using *R* version 4.3.0. Counts and percentages were obtained for all variables, which have been categorized where necessary. For indicator variables to be used in LCA, “At/near restaurants/hawker centers” and “At/near bars/clubs” were merged as “a/near food and beverage establishments,” as the latter was made up of less than 10% of the sample. These variables were dropped from analysis for the same reason – home: “Other areas in your home” and “Don’t know/Not sure”; outside home: “At/near schools/childcare centers/kindergartens/universities,” “At/near hospitals/clinics/polyclinics,” “At/near public parks/playgrounds,” “At/near religious venues,” “At/near community centers/facilities,” “In other persons’ vehicle,” “Others” and “Don’t know/Not sure.”

Analysis was done in two stages. First, LCA was applied to examine the structure underlying the items measuring where smokers chose to smoke, where each individual is classified into their most likely class based on their response patterns on the items related to where they smoke.^21,22^ Our model-building strategy involved starting with the most parsimonious one-class model (“all smokers the same”) and fitting successive models with an increasing number of latent classes to determine the most parsimonious model that provided an adequate fit to the data when compared to the others. The goodness of fit of various models was evaluated using the Akaike information criteria (AIC) and Bayesian information criteria, a global fit index that combines goodness of fit and parsimony. In a comparison of models with the same set of data, models with lower values of the fit indices are preferred. The resulting subgroups or clusters (latent classes) are qualitatively different and assumed to be mutually independent, where members within each latent class differ from another only because of measurement error.^21,23^ LCA was performed using the “poLCA” package in R^24^.

Second, to learn more about the LCA-derived subgroups of Singapore smokers, individuals were assigned to classes based on the estimated modal posterior probabilities of class membership given their observed cigarette smoking patterns. These class assignments were then treated as nominal outcomes and analyzed using multinomial logistic regression. Bivariate multinomial logistic regression was first performed to determine the individual associations of all the covariates with class assignment. Those that were determined to be borderline significant (*p*-value < 0.1) were then included in the multivariable multinomial logistic regression model in order to prevent false negatives when determining the covariates that were associated with class assignment. Multinomial logistic regression was performed using the “nnet” package in R^25^.

## Results

Out of the 1,546 records retained for analysis (Table 1), majority are ethnic Chinese (63.3%), males (77.4%), aged between 25 to 44 years old (inclusive, 54.3%), live in 4-room public housing flats (38.7%) and have minimally pre-university education (36.7%).

**Table 1.** Latent class analysis model diagnostics

Number of latent classes estimated	Estimated population share of smallest class	AIC	BIC	Entropy
2	0.1987	16931.63	17054.52	5.46
3	0.1019	16703.74	16890.76	5.39
4	0.0415	16631.38	16882.53	5.35

Majority were daily smokers (77.1%), and 16.8% were classified as hardcore smokers. Just over half (56.3%) reported smoking both at home and outside home, over one in three smoked only outside the home (35.8%) and less than one in ten reported smoking only at home (7.8%). In total, 92.1% of smokers in our sample reported smoking outside the home, whether exclusively or in combination with smoking at home. Within the home, the most commonly reported location where smokers smoked was the toilet (32.9%), followed by the kitchen (20.8%). Outside the home, the most commonly reported locations where smokers smoked were at/near their workplace (55.6%), followed by the pavements and sidewalks (30.3%) and in public areas of public housing estates (26.9%).

### Latent Class Analysis

Based on the identified list of smoking locations, the latent class models were fit to the data starting with one-class model, which is the most parsimonious, i.e. all smokers smoke in the same places, and followed up by increasing the number of latent classes to a four-class model. The goodness of fit of all models is presented in Table 1. Fit index for AIC improved with an increasing number of classes, but this was not true for BIC, which started increasing when the number of latent classes increased beyond four (Table 1). However, in the four-class model, the proportion of the smallest class was only 4.2% of the sample, which may be too small to be meaningful and result in unnecessary model complexity. Furthermore, in the four-class model, the distribution of responses suggests the smallest class was a subset of another, which violates the assumption that LCA-subgroups should be independent of each other. Hence the more parsimonious three-class model of smoking locations was chosen.

**Figure 2.**
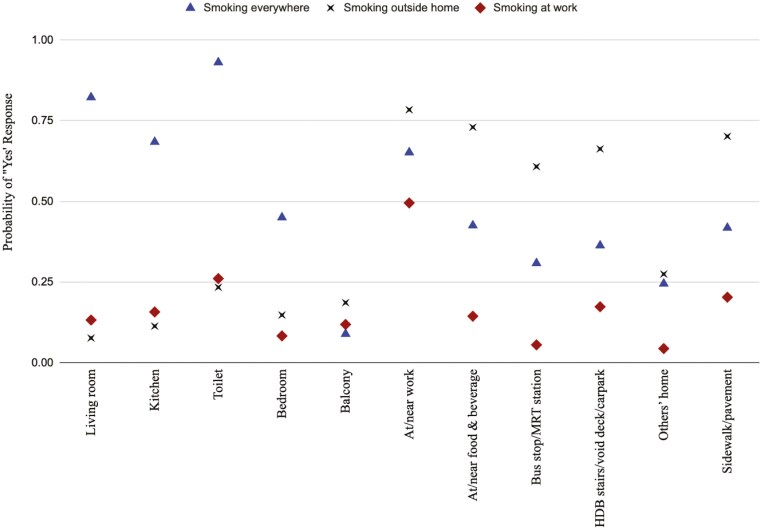
Probability of a “Yes” response to smoking in specific locations in 3-class model.

The estimated probabilities of reporting smoking in different locations in each latent class are displayed graphically in Figure 2. Based on their response profiles, one group could be termed as those who “smoking everywhere” (10.2%), which has high response probabilities for smoking locations both inside home and moderately high response probabilities for locations outside home. In particular, this group is very likely to have reported smoking at home in the toilet and the living room. One group has much noticeably higher response probabilities for most smoking locations outside home than inside home, and can thus be termed as “smoking outside home.” In particular, they had high probabilities of reporting having smoked at or near their workplace, food and beverage establishments, and the sidewalk/pavements. The last group has similar response probabilities to all smoking locations, with the exception of “at/near work,” where a peak can be observed in Figure 2. Hence, this group can be termed “smoking at work.”

The characteristics of the three latent classes are tabulated in Table S1.

### Multinomial Logistic Regression

A bivariate analysis (Table S2) was first carried out to investigate the individual associations between covariates and LCA class assignment. Of the 17 covariates considered, 13 had significant associations with *p*-value < 0.05 and three were borderline significant with 0.05 < *p*-value < 0.10. All were added to the final multivariate model to avoid false negatives.

In the multivariable model (Table S3), seven covariates remained a statistically significant (*p*-value < 0.05) predictor of class membership. Malay smokers were significantly more likely to be assigned “smoking everywhere” than “smoking at work” (OR: 2.102; 95% CI: 1.301 to 3.398) than Chinese smokers. There were no significant differences observed for Indians when compared to Chinese. Smokers living in higher-value housing were significantly less likely to be “smoking everywhere” than “smoking at work” (highest value housing category vs lowest value housing category OR: 0.106; 95% CI: 0.037 to 0.310). Compared to daily smokers, occasional smokers were less likely to be “smoking outside home” (OR: 0.396; 95% CI: 0.250 to 0.628) or “smoking everywhere” (OR: 0.287; 95% CI: 0.132 to 0.623) compared to smoking at work. Hardcore smokers were significantly more likely to be “smoking outside home” (OR: 1.560; 95% CI: 1.028 to 2.369) or “smoking everywhere” (OR: 2.081; 95% CI: 1.355 to 3.197) compared to smoking at work. Smokers who smoked their first cigarette of the day more than 30 minutes of waking i.e., displaying lower nicotine dependency were significantly less likely to be assigned “smoking everywhere” than “smoking at work” (OR: 0.368; 95% CI: 0.233 to 0.582). Smokers who perceive their family norms to not be supportive of their smoking habits were significantly more likely to be assigned “smoking outside home” than “smoking at work” (OR: 3.387; 95% CI: 1.196 to 9.590). See the supplementary material (Tables S3 and S4) for detailed data.

## Discussion

This study highlights the heterogeneity of smoking behaviors in Singapore, identifying three distinct subgroups of smokers based on their preferred smoking locations: “smoke at work” (75.7 %), “smoke outside home” (14.0 %), and “smoke everywhere” (10.2 %). These findings indicate the diverse patterns of smoking in Singapore’s unique multi-ethnic and urban context, offering insights for targeted interventions.

Despite widespread awareness of the harms of secondhand smoke (SHSe), many smokers in Singapore continue to smoke at home (7.8 % exclusively at home, and 56.3 % both at home and outside). This could be reflected in the fact that the most popular location for smoking at home is the toilet (32.9% reported smoking there). However, almost one in five of the smokers also report smoking in the living room, and a similar proportion reports smoking in the kitchen, which are both communal areas which would put other members of the household at risk to SHSe. Although there are strict regulations that ban smoking in public areas of public housing estates (where the vast majority of people in Singapore live), there are no smoking regulations within the confines of private homes. Thus, smoking at home remains an issue in Singapore, and more could be done to promote smoke-free homes, particularly given that more than 80% of the population live in multi-unit apartment buildings, which are at greater risk to SHSe.^26–29^ There would appear to be strong public support for this as well, given the media and political attention given to complaints of SHSe from neighbors, particularly in public housing estates.^30,31^

Among all smokers, 92.1% reported smoking outside the home—either exclusively (35.8%) or in combination with smoking at home (56.3%)—while 64.1% reported smoking at home. Despite this, the LCA model identified “Smoke at Work” as the largest class (75.7%), highlighting the workplace as a key smoking environment. This suggests that workplace-adjacent areas remain a central smoking location for many individuals, underscoring the need for targeted interventions beyond indoor bans. Considering that nearly all indoor spaces are smoke-free and many workplaces—particularly government agencies, educational and healthcare institutions, and indoor food and beverage settings—already have smoking bans in place, many smokers continue to smoke in designated outdoor areas near their workplaces. This presents an opportunity to develop interventions at the workplace setting beyond the existing smoking bans within, as evidence suggests such bans often displace smokers to high-footfall areas, increasing SHSe exposure.^32^ Expanding smoke-free zones, restricting designated smoking areas, and promoting employer-led cessation programs could be effective in reducing SHSe and encouraging smoking cessation. However, it is still important to note that workplace smoking bans have been found to contribute to decreasing smoking prevalence and also SHSe,^33,34^ in particular complete bans that do not make provisions for smokers within the workplace.^35,36^

In the subgroup “smoking outside home,” smokers are almost three times more likely to not report that their family members approve of their smoking habit, and almost twice as likely to be an occasional smoker rather than a daily smoker. This suggests that this group of smokers are still susceptible to the influence of social and familial norms, and may be choosing to smoke outside home due to familial pressures, which is likely protecting their family members from SHSe. This also suggests that using a family-based or community-based approaches^37^ to smoking cessation, such as implementing smoke-free homes, may play a significant role in reducing SHSe at home. This is aligned with existing studies that have shown smoke-free homes are significantly associated with increased smoking cessation amongst smokers and reduced cigarette consumption in adult smokers.^38,39^

The results of the current study suggest that hardcore smokers would most likely to be exposing others to SHS in a variety of locations, as were daily smokers. This finding is aligned with existing evidence indicating that those who displayed greater levels of nicotine dependence were more likely to smoke in many different places, while those that had lower levels of nicotine dependence displayed characteristics that were more aligned to social smokers who smoked only at specific locations and social contexts.^7^ Given the behaviors displayed by hardcore smokers in Singapore, there could be interventions that can be explored to further lower their nicotine dependency. Interventions could focus on decreasing nicotine dependency and the number of cigarettes smoked per day in these smokers. This could be done through individual interventions such as motivational interviewing,^40^ or more broad-based approaches such as regulating the amount of nicotine content in cigarettes.^41,42^

In the study sample, Malay smokers had significantly higher odds of being placed in the “smoking everywhere” subgroup. This is consistent with the fact that smoking prevalence in the Malay community is disproportionately high amongst the three major ethnic groups in Singapore.^43^ The Malay community has stronger social ties and ethnic identity than other ethnic groups in Singapore,^44–46^ and this could provide a lever for implementation of smoking cessation interventions. Further, it was found that peers’ disapproval had significantly lowered the odds of Malay smokers from being classified as a hardcore smoker,^47^ lending further support to tailored interventions for the Malay community. There is evidence that the Malay-Muslim community’s health behavior is influenced by specific cultural, traditional, spiritual/religious, and intergenerational beliefs.^48,49^ Future studies could further look into developing targeted interventions for this particular group that is already at a disadvantage in terms of tobacco-related health disparities, taking the above listed into consideration.

While Singapore has implemented advanced tobacco control policies and regulations in a unique urban and multi-ethnic context, the differences observed in this study offer valuable insights for addressing the tobacco epidemic globally. These findings underscore the potential of community-based approaches that harness familial, spiritual/religious, cultural, and intergenerational beliefs to create effective tobacco control strategies.^3,14,37,50^ Such approaches could be particularly relevant for countries seeking to adapt interventions to their own social and cultural contexts.

The main strength of our study lies in its unique characterization of smoking behavior within a multi-ethnic, urban Asian setting, employing a novel approach compared to existing research. By utilizing LCA, we were able to identify heterogeneous smoker subgroups, supported by a sufficiently large sample size that allowed for meaningful analysis. Furthermore, our dataset included a substantial number of participants from Malay and Indian minorities, providing valuable insights into the role of ethnicity in shaping smoking behaviors—a critical contribution in the context of Singapore’s diverse population.

However, several limitations should be taken into account when interpreting these findings. First, as the participants were drawn from existing studies, the sample may not fully represent the broader smoker population in Singapore, which could affect the generalizability of our results. Second, the reliance on self-reported data introduces the possibility of inaccuracies due to social desirability bias and recall bias. Third, the cross-sectional nature of the study restricts our ability to establish causality or examine temporal trends in smoking behavior. Additionally, the reported smoking habits may have been influenced by the unique circumstances of the COVID-19 pandemic and associated lockdown measures during the study period. Finally, while “smoke at work” was selected as the reference group in the multinomial logistic regression analysis due to its large size and ease of interpretability, employment status was not explicitly accounted for, which could have influenced the results. Despite this limitation, the choice of this reference group offers practical advantages for analyses aimed at informing policy decisions, as it aligns with workplace smoking interventions and broader tobacco control strategies.

## Conclusion

Using survey data on the preferred smoking locations of smokers in Singapore, this study identified three distinct latent classes: those who smoke predominantly at work, those who smoke mostly outside their homes, and a group who smoke everywhere. This observed heterogeneity in smokers’ choice of smoking locations highlights opportunities for targeted interventions aimed at both smoking cessation and protecting nonsmokers from SHSe. Tailored interventions that address the specific characteristics of each subgroup could further enhance smoking cessation efforts and protect nonsmokers from exposure. Findings from the study suggest that familial influences and pressure are key factors in whether a smoker chooses to smoke outside of home rather than at work, indicating that social norms play a crucial role in shaping smoking behavior. This lends support to social norm change strategies, such as promoting smoke-free home policies and community-based interventions, as potential means to reduce SHSe in domestic settings. Finally, targeted efforts are needed to support smoking cessation in the Malay community in Singapore, as their higher likelihood of being in the “smoking everywhere” subgroup increases their risk of tobacco-related health disparities. Culturally tailored interventions addressing their unique social and familial contexts could help reduce these disparities and improve health outcomes.

## Supplementary Material

ntaf076_suppl_Supplementary_Materials

## Data Availability

Data are available from the corresponding author, upon reasonable request.
