# Supplementary material for: Where Do Smokers in Singapore Smoke? A Latent Class Analysis to Classify Smokers Based On Smoking Location
Source: Nicotine Tob Res. 2025 Apr 9;27(12):2297–304. doi: 10.1093/ntr/ntaf076 (PMC12641174; doi:10.1093/ntr/ntaf076)
Supplement: ntaf076_suppl_Supplementary_Materials [file ntaf076_suppl_supplementary_materials.docx]

|  | **All smokers** | | **“Smoke at work”** | **“Smoke outside home”** | **“Smoke everywhere”** | ***p*-value*** |
| --- | --- | --- | --- | --- | --- | --- |
|  | **No. (%)** | | **No. (%)** | **No. (%)** | **No. (%)** |  |
|  | 1546 (100.0%) | | 1171 (75.7%) | 217 (14.0%) | 158 (10.2%) |  |
| **Sociodemographics** |  |  | | | |  |
| Gender |  |  | | | | 0.022 |
| *Male* | 1196 (77.4%) | | 904 (75.6%) | 180 (15.1%) | 112 (9.4%) |  |
| *Female* | 350 (22.6%) | | 267 (76.3%) | 37 (10.6%) | 46 (13.1%) |  |
| Age |  |  | | | | 0.002 |
| *19 - 24 years old* | 149 (9.6%) | | 104 (69.8%) | 36 (24.2%) | 9 (6.0%) |  |
| *25 - 44 years old* | 839 (54.3%) | | 631 (75.2%) | 119 (14.2%) | 89 (10.6%) |  |
| *45 - 64 years old* | 513 (33.2%) | | 396 (77.2%) | 60 (11.7%) | 57 (11.1%) |  |
| *65 years old and above* | 45 (2.9%) | | 40 (88.9%) | 2 (4.4%) | 3 (6.7%) |  |
| Ethnicity |  |  | | | | < 0.001 |
| *Chinese* | 978 (63.3%) | | 746 (76.3%) | 152 (15.5%) | 80 (8.2%) |  |
| *Indian* | 212 (13.7%) | | 174 (82.1%) | 24 (11.3%) | 14 (6.6%) |  |
| *Malay* | 356 (23.0%) | | 251 (70.5%) | 41 (11.5%) | 64 (18.0%) |  |
| Highest education attainment |  | |  |  | | < 0.001 |
| *Primary and below* | 104 (6.7%) | | 76 (73.1%) | 10 (9.6%) | 18 (17.3%) |  |
| *Secondary* | 515 (33.3%) | | 383 (74.4%) | 54 (10.5%) | 78 (15.1%) |  |
| *Pre-university* | 568 (36.7%) | | 422 (74.3%) | 97 (17.1%) | 49 (8.6%) |  |
| *University and above* | 359 (23.2%) | | 290 (80.8%) | 56 (15.6%) | 13 (3.6%) |  |
| Current marital status |  |  | | | | < 0.001 |
| *Single* | 510 (33.0%) | | 361 (70.8%) | 103 (20.2%) | 46 (9.0%) |  |
| *Married* | 882 (57.1%) | | 700 (79.4%) | 95 (10.8%) | 87 (9.9%) |  |
| *Widowed/Divorced/Separated* | 154 (10.0%) | | 110 (71.4%) | 19 (12.3%) | 25 (16.2%) |  |
| Housing type |  | |  |  | | < 0.001 |
| *HDB 1-2 room* | 144 (9.3%) | | 93 (64.6%) | 14 (9.7%) | 37 (25.7%) |  |
| *HDB 3 room* | 297 (19.2%) | | 225 (75.8%) | 38 (12.8%) | 34 (11.4%) |  |
| *HDB 4 room* | 599 (38.7%) | | 451 (%) | 87 (14.5%) | 61 (10.2%) |  |
| *HDB 5 room/Executive flat* | 390 (25.2%) | | 308 (79.0%) | 60 (15.4%) | 22 (5.6%) |  |
| *Condo/Landed/Others* | 116 (7.5%) | | 94 (81.0%) | 18 (15.5%) | 4 (3.4%) |  |
| **Smoking-related characteristics** |  |  | | | |  |
| Smoking Status |  | |  |  |  | < 0.001 |
| *Daily* | 1192 (77.1%) | | 855 (71.7%) | 187 (15.7%) | 150 (12.6%) |  |
| *Occasional* | 354 (22.9%) | | 316 (8.5%) | 30 (13.8%) | 8 (2.3%) |  |
|  |  | |  |  |  |  |
| Hardcore smoking indicator |  | |  |  |  | < 0.001 |
| *Yes* | 259 (16.8%) | | 157 (60.6%) | 49 (18.9%) | 105 (8.2%) |  |
| *No* | 1287 (83.2%) | | 1014 (78.8%) | 168 (13.1%) | 53 (20.5%) |  |
| How soon after you wake up do you usually have your first smoke? |  |  | | | | < 0.001 |
| *≤ 30 minutes* | 803 (51.9%) | | 547 (68.1%) | 127 (15.8%) | 129 (16.1%) |  |
| *> 30 minutes* | 743 (48.1%) | | 624 (84.0%) | 90 (12.1%) | 29 (3.9%) |  |
| Reported smoking location |  | |  |  |  | < 0.001 |
| *Home only* | 121 (7.8%) | | 113 (93.4%) | 0 (0.0%) | 8 (6.6%) |  |
| *Home and outside home* | 871 (56.3%) | | 587 (67.4%) | 134 (15.4%) | 150 (17.2%) |  |
| *Outside home only* | 554 (35.8%) | | 471 (85.0%) | 83 (15.0%) | 0 (0.0%) |  |
| Smoking locations reported at home |  | |  |  |  |  |
| *Living room* | 307 (19.9%) | | 150 (48.9%) | 10 (3.3%) | 147 (47.9%) | < 0.001 |
| *Kitchen* | 321 (20.8%) | | 183 (57.0%) | 24 (7.5%) | 114 (35.5%) | < 0.001 |
| *Toilet* | 509 (32.9%) | | 301 (59.1%) | 57 (11.2%) | 151 (29.7%) | < 0.001 |
| *Bedroom* | 205 (13.3%) | | 94 (45.9%) | 36 (17.6%) | 75 (36.6%) | < 0.001 |
| *Balcony* | 194 (12.5%) | | 138 (71.1%) | 42 (21.6%) | 14 (7.2%) | 0.003 |
| Smoking locations reported outside home |  | |  |  |  |  |
| *At/near work* | 860 (55.6%) | | 581 (67.6%) | 177 (20.6%) | 102 (11.9%) | < 0.001 |
| *At/near food & beverage* | 409 (26.5%) | | 174 (42.5%) | 167 (40.8%) | 68 (16.6%) | < 0.001 |
| *Bus stop/MRT station* | 259 (16.8%) | | 52 (20.1%) | 157 (60.6%) | 50 (19.3%) | < 0.001 |
| *HDB stairs/void deck/carpark* | 416 (26.9%) | | 207 (49.8%) | 148 (35.6%) | 61 (14.7%) | < 0.001 |
| *Others’ home* | 156 (10.1%) | | 51 (32.7%) | 63 (40.4%) | 42 (26.9%) | < 0.001 |
| *Sidewalk/pavement* | 468 (30.3%) | | 237 (50.6%) | 161 (34.4%) | 70 (15.0%) | < 0.001 |
| In the past month, have you talked with your family members about your desire to quit smoking? |  |  | | | | 0.001 |
| *Yes* | 257 (16.6%) | | 209 (81.3%) | 26 (10.1%) | 22 (8.6%) |  |
| *No/not sure* | 1289 (83.4%) | | 962 (74.6%) | 191 (14.8%) | 136 (10.6%) |  |
| In the past month, have you talked with your friends about your desire to quit smoking? |  |  | | | | 0.002 |
| *Yes* | 335 (21.7%) | | 267 (79.7%) | 45 (13.4%) | 23 (6.9%) |  |
| *No/not sure* | 1211 (78.3%) | | 904 (74.6%) | 172 (14.2%) | 135 (11.1%) |  |
| How do you think your friends in general feel / would feel about you smoking? |  |  | | | | 0.684 |
| *Approve* | 138 (8.9%) | | 104 (75.4%) | 20 (14.5%) | 14 (10.1%) |  |
| *Neutral/Disapprove* | 1408 (91.1%) | | 1067 (75.8%) | 197 (14.0%) | 144 (10.2%) |  |
| How do you think your family members (e.g. parents, siblings) feel/would feel about you smoking? |  |  | | | | < 0.001 |
| *Approve* | 89 (5.8%) | | 71 (79.8%) | 4 (4.5%) | 14 (15.7%) |  |
| *Neutral/Disapprove* | 1457 (94.2%) | | 1100 (75.5%) | 213 (14.6%) | 144 (9.9%) |  |
| How many of your closest friends (the ones you hang out with the most) use tobacco? |  |  | | | | < 0.001 |
| *None or <half* | 526 (34.0%) | | 426 (81.0%) | 64 (12.2%) | 36 (6.8%) |  |
| *Approx half* | 450 (29.1%) | | 344 (76.4%) | 60 (13.3%) | 46 (10.2%) |  |
| *Most or all* | 570 (36.9%) | | 401 (70.4%) | 93 (16.3%) | 76 (13.3%) |  |
| **Lifestyle and health status** |  |  | | | |  |
| Consumption of alcohol |  |  | | | | < 0.001 |
| *Non-drinker* | 593 (38.4%) | | 457 (77.1%) | 62 (10.5%) | 74 (12.5%) |  |
| *Occasional drinker* | 628 (40.6%) | | 477 (76.0%) | 93 (14.8%) | 58 (9.2%) |  |
| *Frequent drinker* | 249 (16.1%) | | 183 (73.5%) | 51 (20.5%) | 15 (6.0%) |  |
| *Regular drinker* | 76 (4.9%) | | 54 (71.1%) | 11 (14.5%) | 11 (14.5%) |  |
| Amount of weekly physical activities |  |  | | | | 0.046 |
| *None* | 358 (23.2%) | | 268 (74.2%) | 39 (10.9%) | 51 (14.2%) |  |
| *1-2 times* | 759 (49.1%) | | 583 (76.8%) | 108 (14.2%) | 68 (9.0%) |  |
| *3-6 times* | 345 (22.3%) | | 256 (74.2%) | 59 (17.1%) | 30 (8.7%) |  |
| *Daily* | 84 (5.4%) | | 64 (76.2%) | 11 (13.1%) | 9 (10.7%) |  |
| Self-perceived health status |  |  | | | | 0.077 |
| *Excellent* | 46 (3.0%) | | 38 (82.6%) | 5 (10.9%) | 3 (6.5%) |  |
| *Very good* | 224 (14.5%) | | 173 (77.2%) | 37 (16.5%) | 14 (6.2%) |  |
| *Good* | 690 (44.6%) | | 537 (77.8%) | 80 (11.6%) | 73 (10.6%) |  |
| *Fair* | 522 (33.8%) | | 380 (72.8%) | 83 (15.9%) | 59 (11.3%) |  |
| *Poor* | 64 (4.1%) | | 43 (67.2%) | 12 (18.8%) | 9 (14.1%) |  |

^#^Almost 80% of Singapore population lives in public housing known as HDB (Housing Development Board) flats, which are rented or bought from the government, with eligibility and the amount subsidised determined by household income, and size of apartment sought.

*Chi-square test of independence. Fisher’s test when cell count ≤5

#### Supplementary Table 1. Individual characteristics of smokers by latent class of location of smoking

|  |  | **“smoking at work” vs “smoking outside home”** | | | **“smoking at work” vs “smoking everywhere”** | | | ***p*-value** |
| --- | --- | --- | --- | --- | --- | --- | --- | --- |
|  |  | **Odds Ratio** | **(95% CI)** | | **Odds Ratio** | **(95% CI)** | |  |
| **Sociodemographics** |  |  |  |  |  |  |  |  |
| Gender |  |  |  |  |  |  |  |  |
|  | *Female* | reference | | | reference | | | <0.001 |
|  | *male* | 1.436 | (0.984 – 2.097) | | 0.719 | (0.497 – 1.039) | |  |
| Age |  |  |  |  |  |  |  |  |
|  | *18-24* | reference | | | reference | | | 0.021 |
|  | *25-44* | 0.545 | (0.355 – 0.836) | | 1.629 | (0.797 – 3.331) | |  |
|  | *45-64* | 0.438 | (0.275 – 0.698) | | 1.664 | (0.798 – 3.469) | |  |
|  | *>64* | 0.145 | (0.033 – 0.630) | | 0.866 | (0.223 – 3.361) | |  |
| Ethnicity |  |  |  |  |  |  |  |  |
|  | *Chinese* | reference | | | reference | | | <0.001 |
|  | *Indian* | 0.677 | (0.427 – 1.073) | | 0.750 | (0.415 – 1.355) | |  |
|  | *Malay* | 0.802 | (0.551 – 1.166) | | 2.377 | (1.661 – 3.403) | |  |
| Highest education attainment |  |  |  |  |  |  |  |  |
|  | *Primary* | reference | | | reference | | | <0.001 |
|  | *Secondary* | 1.073 | (0.523 – 2.203) | | 0.860 | (0.487 – 1.518) | |  |
|  | *Pre-university* | 1.749 | (0.872 – 3.507) | | 0.490 | (0.271 – 0.886) | |  |
|  | *University and above* | 1.470 | (0.716 – 3.017) | | 0.188 | (0.088 – 0.401) | |  |
| Current marital status |  |  |  |  |  |  |  |  |
|  | *Married* | reference | | | reference | | | <0.001 |
|  | *Single* | 2.102 | (1.548 – 2.854) | | 1.026 | (0.703 – 1.498) | |  |
|  | *Widowed/divorced/separated* | 1.273 | (0.748 – 2.164) | | 1.828 | (1.122 – 2.977) | |  |
| Housing type |  |  |  |  |  |  |  |  |
|  | *1-2 room* | reference | | | reference | | | <0.001 |
|  | *3 room* | 1.122 | (0.581 – 2.167) | | 0.380 | (0.225 – 0.642) | |  |
|  | *4 room* | 1.281 | (0.698 – 2.353) | | 0.340 | (0.213 – 0.540) | |  |
|  | *5 room/EC* | 1.294 | (0.691 – 2.423) | | 0.179 | (0.101 – 0.319) | |  |
|  | *Condo/Landed/Others* | 1.273 | (0.598 – 2.706) | | 0.106 | (0.037 – 0.310) | |  |
| **Smoking-related characteristics** | |  |  |  |  |  |  |  |
| Smoking status |  |  |  |  |  |  |  |  |
|  | *Daily* | reference | | | reference | | | <0.001 |
|  | *Occasional* | 0.434 | (0.289 – 0.651) | | 0.144 | (0.070 – 0.296) | |  |
| Hardcore smoking indicator | |  |  |  |  |  |  |  |
|  | *No* | reference | | | reference | | | <0.001 |
|  | *Yes* | 1.883 | (1.313 – 2.701) | | 3.254 | (2.247 – 4.714) | |  |
| How soon after you wake up do you usually have your first smoke? | |  |  |  |  |  |  |  |
|  | *≤ 30 minutes* | reference | | | reference | | |  |
|  | *> 30 minutes* | 0.621 | (0.463 – 0.834) | | 0.198 | (0.130 – 0.301) | | <0.001 |
| In the past month, have you talked with your family members about your desire to quit smoking? | |  |  |  |  |  |  |  |
|  | *Yes* | reference | | | reference | | | 0.056 |
|  | *No/not sure* | 1.595 | (1.032 – 2.465) | | 1.344 | (0.837 – 2.160) | |  |
| In the past month, have you talked with your friends about your desire to quit smoking? | |  |  |  |  |  |  |  |
|  | *Yes* | reference | | | reference | | | 0.046 |
|  | *No/not sure* | 1.129 | (0.792 – 1.609) | | 1.733 | (1.091 – 2.753) | |  |
| How do you think your family members (e.g. parents, siblings) feel/would feel about you smoking? | |  |  |  |  |  |  |  |
|  | *Approve* | reference | | | reference | | | <0.001 |
|  | *Neutral/Disapprove* | 3.421 | (1.237 – 9.462) | | 0.664 | (0.364 – 1.209) | |  |
| How do you think your friends in general feel / would feel about you smoking? | |  |  |  |  |  |  |  |
|  | *Approve* | reference | | | reference | | | 0.987 |
|  | *Neutral/Disapprove* | 0.960 | (0.581 – 1.586) | | 1.003 | (0.559 – 1.798) | |  |
| How many of your closest friends (the ones you hang out with the most) use tobacco? | |  |  |  |  |  |  |  |
|  | *None or <half* | reference | | | reference | | | <0.001 |
|  | *Approx half* | 1.161 | (0.794 – 1.698) | | 1.582 | (1.000 – 2.503) | |  |
|  | *Most or all* | 1.543 | (1.091 – 2.183) | | 2.243 | (1.475 – 3.412) | |  |
| **Lifestyle and health status** |  |  |  |  |  |  |  |  |
| Consumption of alcohol | |  |  |  |  |  |  |  |
|  | *Non-drinker* | reference | | | reference | | | <0.001 |
|  | *Occasional drinker* | 1.438 | (1.016 – 2.034) | | 0.751 | (0.521 – 1.084) | |  |
|  | *Frequent drinker* | 2.054 | (1.367 – 3.088) | | 0.506 | (0.283 – 0.904) | |  |
|  | *Regular drinker* | 1.501 | (0.745 – 3.021) | | 1.257 | (0.628 – 2.516) | |  |
| Amount of weekly physical activities | |  |  |  |  |  |  |  |
|  | *Daily* | reference | | | reference | | | 0.055 |
|  | *None* | 0.846 | (0.411 – 1.744) | | 1.351 | (0.633 – 2.885) | |  |
|  | *1-2 times* | 1.077 | (0.550 – 2.110) | | 0.828 | (0.395 – 1.737) | |  |
|  | *3-6 times* | 1.340 | (0.666 – 2.699) | | 0.831 | (0.376 – 1.838) | |  |
|  |  |  |  | |  |  | |  |
| Self-perceived health status | |  |  |  |  |  |  |  |
|  | *Excellent* | reference | | | reference | | | 0.064 |
|  | *Very good* | 1.626 | (0.600 – 4.409) | | 1.025 | (0.281 – 3.744) | |  |
|  | *Good* | 1.132 | (0.432 – 2.963) | | 1.721 | (0.519 – 5.712) | |  |
|  | *Fair* | 1.660 | (0.634 – 4.346) | | 1.966 | (0.588 – 6.576) | |  |
|  | *Poor* | 2.121 | (0.685 – 6.573) | | 2.651 | (0.668 – 10.516) | |  |

####

#### Supplementary Table 2. Bivariate multinomial logistic regression comparing “smoking at work” to “smoking everywhere” and “smoking outside home”

|  |  | **“smoking at work” vs “smoking outside home”** | | ***p*-value** | **“smoking at work” vs “smoking everywhere”** | | ***p*-value** |
| --- | --- | --- | --- | --- | --- | --- | --- |
|  |  | **Odds Ratio** | **(95% CI)** |  | **Odds Ratio** | **(95% CI)** |  |
| **Sociodemographics** | |  |  |  |  |  |  |
| Gender | |  |  |  |  |  |  |
|  | *Female* | reference | |  | reference | | 0.126 |
|  | *Male* | 1.394 | (0.923 – 2.103) | 0.113 | 0.802 | (0.520 – 1.236) | 0.318 |
| Age | |  |  |  |  |  |  |
|  | *18-24* | reference | |  | reference | | 0.154 |
|  | *25-44* | 0.639 | (0.382 – 1.070) | 0.088 | 1.101 | (0.485 – 2.499) | 0.817 |
|  | *45-64* | 0.543 | (0.294 – 1.001) | 0.051 | 0.921 | (0.376 – 2.255) | 0.857 |
|  | *>64* | 0.192 | (0.040 – 0.918) | 0.039 | 0.379 | (0.082 – 1.749) | 0.213 |
| Ethnicity | |  |  |  |  |  |  |
|  | *Chinese* | reference | |  | reference | | 0.001 |
|  | *Indian* | 0.634 | (0.388 – 1.035) | 0.068 | 0.616 | (0.327 – 1.163) | 0.135 |
|  | *Malay* | 1.175 | (0.735 – 1.877) | 0.500 | 2.102 | (1.301 – 3.398) | 0.002 |
| Highest education attainment | |  |  |  |  |  |  |
|  | *Primary* | reference | |  | reference | | 0.195 |
|  | *Secondary* | 0.971 | (0.457 – 2.061) | 0.939 | 0.990 | (0.523 – 1.875) | 0.975 |
|  | *Pre-university* | 1.289 | (0.598 – 2.780) | 0.516 | 0.826 | (0.405 – 1.686) | 0.599 |
|  | *University and above* | 1.196 | (0.529 – 2.703) | 0.667 | 0.438 | (0.180 – 1.066) | 0.069 |
| Current marital status | |  |  |  |  |  |  |
|  | *Married* | reference | |  | reference | | 0.022 |
|  | *Single* | 1.853 | (1.270 – 2.705) | 0.001 | 1.436 | (0.904 – 2.281) | 0.125 |
|  | *Widowed/divorced/separated* | 1.353 | (0.769 – 2.379) | 0.294 | 1.225 | (0.699 – 2.146) | 0.477 |
| Housing type | |  |  |  |  |  |  |
|  | *1-2 room* | reference | |  | reference | | 0.017 |
|  | *3 room* | 1.043 | (0.519 – 2.096) | 0.905 | 0.510 | (0.281 – 0.925) | 0.026 |
|  | *4 room* | 1.190 | (0.621 – 2.281) | 0.601 | 0.485 | (0.282 – 0.834) | 0.009 |
|  | *5 room/EC* | 1.306 | (0.662 – 2.578) | 0.441 | 0.317 | (0.163 – 0.614) | <0.001 |
|  | *Condo/Landed/Others* | 0.983 | (0.434 – 2.227) | 0.968 | 0.183 | (0.058 – 0.576) | 0.004 |
| **Smoking-related characteristics** | |  |  |  |  |  |  |
| Smoking Status | |  |  |  |  |  |  |
|  | *Daily* | reference | |  | reference | | <0.001 |
|  | *Occasional* | 0.396 | (0.250 – 0.628) | <0.001 | 0.287 | (0.132 – 0.623) | 0.002 |
| Hardcore smoking indicator | |  |  |  |  |  |  |
|  | *No* | reference | |  | reference | | 0.001 |
|  | *Yes* | 1.560 | (1.028 – 2.369) | 0.036 | 2.081 | (1.355 – 3.197) | <0.001 |
| How soon after you wake up do you usually have your first smoke? | |  |  |  |  |  |  |
|  | *≤ 30 minutes* | reference | |  | reference | | <0.001 |
|  | *> 30 minutes* | 0.726 | (0.517 – 1.019) | 0.065 | 0.368 | (0.233 – 0.582) | <0.001 |
| In the past month, have you talked with your family members about your desire to quit smoking? | |  |  |  |  |  |  |
|  | *Yes* | reference | |  | reference | | 0.888 |
|  | *No/not sure* | 1.143 | (0.664 – 1.968) | 0.630 | 1.001 | (0.533 – 1.882) | 0.998 |
| In the past month, have you talked with your friends about your desire to quit smoking? | |  |  |  |  |  |  |
|  | *Yes* | reference | |  | reference | | 0.598 |
|  | *No/not sure* | 0.976 | (0.622 – 1.532) | 0.917 | 1.350 | (0.737 – 2.474) | 0.332 |
| How do you think your family members (e.g. parents, siblings) feel/would feel about you smoking? | |  |  |  |  |  |  |
|  | *Approve* | reference | |  | reference | | 0.019 |
|  | *Neutral/Disapprove* | 3.387 | (1.196 – 9.590) | 0.022 | 0.868 | (0.439 – 1.716) | 0.683 |
| How many of your closest friends (the ones you hang out with the most) use tobacco? | |  |  |  |  |  |  |
|  | *None or <half* | reference | |  | reference | | 0.531 |
|  | *Approx half* | 1.037 | (0.694 – 1.550) | 0.859 | 1.392 | (0.848 – 2.286) | 0.190 |
|  | *Most or all* | 1.241 | (0.847 – 1.819) | 0.268 | 1.236 | (0.769 – 1.986) | 0.381 |
| **Lifestyle and health status** | |  |  |  |  |  |  |
| Consumption of alcohol | |  |  |  |  |  |  |
|  | *Non-drinker* | reference | |  | reference | | 0.116 |
|  | *Occasional drinker* | 1.430 | (0.946 – 2.163) | 0.089 | 1.346 | (0.857 – 2.112) | 0.197 |
|  | *Frequent drinker* | 2.094 | (1.273 – 3.445) | 0.004 | 1.176 | (0.591 – 2.340) | 0.644 |
|  | *Regular drinker* | 1.332 | (0.613 – 2.896) | 0.469 | 1.480 | (0.670 – 3.267) | 0.332 |
| Amount of weekly physical activities | |  |  |  |  |  |  |
|  | *Daily* | reference | |  | reference | | 0.559 |
|  | *None* | 0.713 | (0.333 – 1.529) | 0.384 | 1.124 | (0.494 – 2.560) | 0.781 |
|  | *1-2 times* | 0.813 | (0.398 – 1.659) | 0.569 | 0.852 | (0.382 – 1.903) | 0.696 |
|  | *3-6 times* | 1.052 | (0.501 – 2.211) | 0.894 | 1.088 | (0.457 – 2.593) | 0.849 |
|  |  |  |  |  |  |  |  |
|  |  |  |  |  |  |  |  |
|  |  |  |  |  |  |  |  |
|  |  |  |  |  |  |  |  |
|  |  |  |  |  |  |  |  |
|  |  |  |  |  |  |  |  |
| Self-perceived health status | |  |  |  |  |  |  |
|  | *Excellent* | reference | |  | reference | | 0.103 |
|  | *Very good* | 1.458 | (0.514 – 4.136) | 0.479 | 0.986 | (0.248 – 3.919) | 0.984 |
|  | *Good* | 1.023 | (0.373 – 2.808) | 0.964 | 1.677 | (0.466 – 6.031) | 0.428 |
|  | *Fair* | 1.660 | (0.604 – 4.565) | 0.327 | 1.598 | (0.442 – 5.782) | 0.475 |
|  | *Poor* | 1.908 | (0.573 – 6.356) | 0.292 | 2.319 | (0.530 – 10.144) | 0.264 |

####

#### Supplementary Table 3. Multivariate multinomial logistic regression comparing “smoking at work” to “smoking everywhere” and “smoking outside home”
